# The roles of neutrophils in cardiovascular diseases

**DOI:** 10.3389/fcvm.2025.1526170

**Published:** 2025-03-19

**Authors:** Yanjie Lian, Xiaolei Lai, Cong Wu, Li Wang, JuJu Shang, Heyi Zhang, Sihan Jia, Wenlong Xing, Hongxu Liu

**Affiliations:** ^1^ Department of Cardiovascular Medicine, Beijing Hospital of Traditional Chinese Medicine, Capital Medical University, Beijing, China; ^2^ Beijing Hospital of Traditional Chinese Medicine, Huairou Hospital, Beijing, China; ^3^ Guang’anmen Hospital, China Academy of Chinese Medical Sciences, Beijing, China

**Keywords:** neutrophils, cardiovascular diseases, neutrophil extracellular traps (NETs), inflammation, innate immunology

## Abstract

The immune response plays a vital role in the development of cardiovascular diseases (CVDs). As a crucial component of the innate immune system, neutrophils are involved in the initial inflammatory response following cardiovascular injury, thereby inducing subsequent damage and promoting recovery. Neutrophils exert their functional effects in tissues through various mechanisms, including activation and the formation of neutrophil extracellular traps (NETs). Once activated, neutrophils are recruited to the site of injury, where they release inflammatory mediators and cytokines. This study discusses the main mechanisms associated with neutrophil activity and proposes potential new therapeutic targets. In this review, we systematically summarize the diverse phenotypes of neutrophils in disease regulatory mechanisms, different modes of cell death, and focus on the relevance of neutrophils to various CVDs, including atherosclerosis, acute coronary syndrome, myocardial ischemia/reperfusion injury, hypertension, atrial fibrillation, heart failure, and viral myocarditis. Finally, we also emphasize the preclinical/clinical translational significance of neutrophil-targeted strategies.

## 1 Introduction

Cardiovascular diseases (CVDs) are currently considered the leading cause of death worldwide, with approximately 50% of annual deaths attributed to CVDs (1). During the past few years, there has been a renewed focus on innate immunology. Neutrophils, the most abundant type of immune cell in the innate immune system (2), serve as a vital defense against pathogens. Traditionally viewed as the final effector cells of acute inflammatory responses and playing a leading role in the clearance of extracellular pathogens, the roles of neutrophils have expanded based on increasing evidence (3–5). They regulate inflammation and the immune system by producing a variety of cytokines and other inflammatory factors (6, 7). Early studies focused on the impact of neutrophils on rheumatic immunity, cancer, sepsis, etc., with a limited understanding of their role in cardiovascular inflammation. As research progresses, the significance of neutrophils in the development of CVDs has been increasingly investigated. Neutrophils are widely involved in the occurrence and development of CVDs; overactivation can exacerbate the progression of various CVDs, including atherosclerosis (8), myocardial infarction (9), hypertension (10), and atrial fibrillation (11). This overview focuses on the basic principles of neutrophil biology associated with cardiovascular pathophysiology and its significance in CVDs.

## 2 Formation, markers, and cardiac biological functions of neutrophils

### 2.1 Formation of neutrophils

Neutrophils are the most abundant type of leukocytes, constituting the crucial first line of defense against infections, the general life cycle of neutrophils includes their formation in the bone marrow, release into the bloodstream, migration to tissues, and eventual clearance (12). They are originated from hematopoietic stem cells (HSCs) in the bone marrow and undergo different stages of differentiation under the regulation of granulocyte colony-stimulating factor (G-CSF). These stages include the progression from multipotent progenitor cells (HPCs), through myeloid lineage-committed progenitors, to common myeloid progenitor cells (CMPs), and granulocyte-monocyte progenitor cells (GMPs). Then, GMPs enter the mitotic pool, where they rapidly divide and differentiate into promyelocytes, myelocytes, subsequently into late promyelocytes, band neutrophils, and finally into segmented nuclear neutrophils (13). Mature segmented nuclear neutrophils are released into the bloodstream through the bone marrow-blood barrier, ready to respond to signals of inflammation or infection (3) The traditional view holds that neutrophils have a half-life of approximately 6–12 h (14). They reside in the vasculature for an average of only 6–8 h before crossing the vessel walls to enter tissues and exert their functions. Once in the tissues, they typically do not return to the bloodstream. Aged neutrophils are usually cleared by apoptosis within 1 day or are phagocytosed by macrophages and other immune cells. This short lifespan has long been considered a limitation for their role in immune regulation. However, studies by Pillay et al. (15) show that the average lifespan of circulating human neutrophils is approximately 5.4 days. This finding challenges the previous notion of a short neutrophil lifespan and provides a new perspective for re-evaluating the functions of neutrophils in health and disease.

### 2.2 Heterogeneity and markers of neutrophils

**Figure 1:**
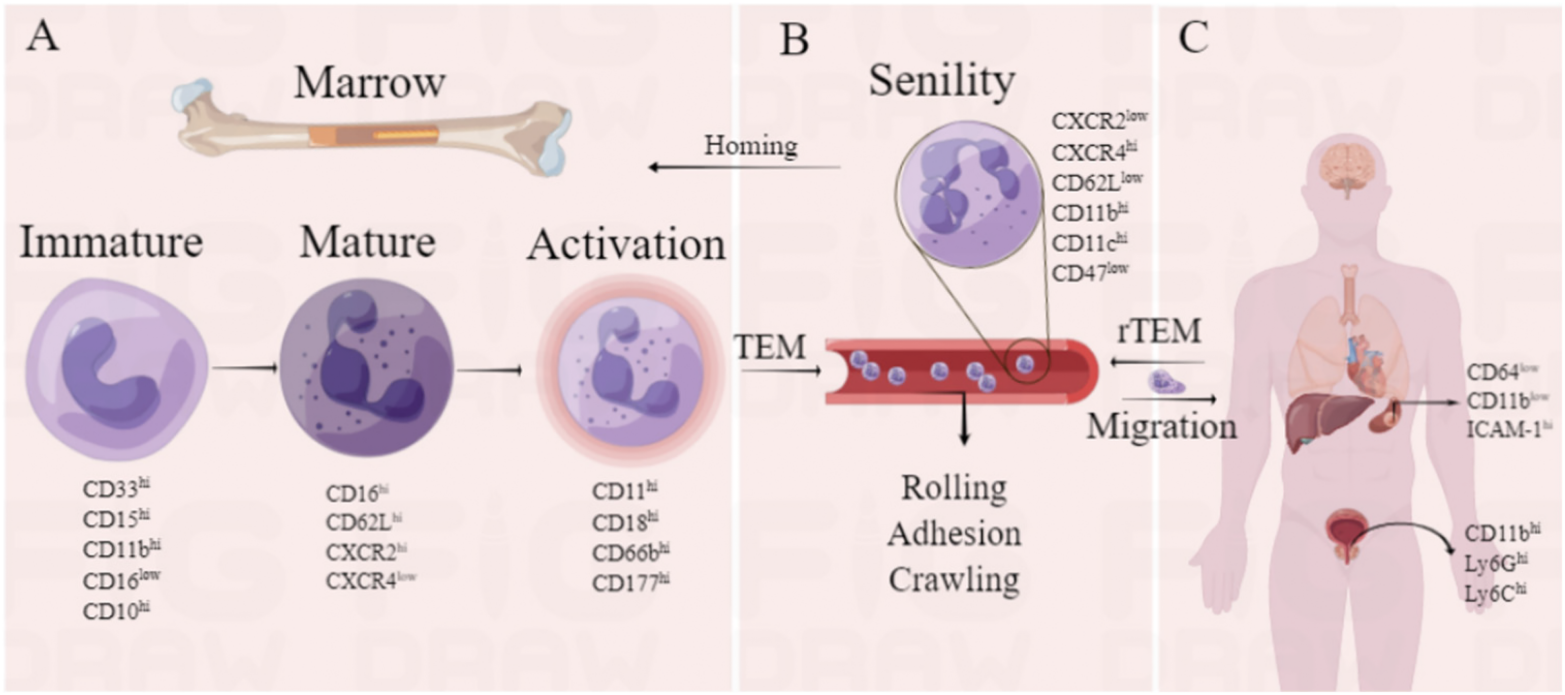
Partial markers of neutrophils. illustrates the heterogeneity of neutrophils in different states, including the markers of immature, mature, and activated neutrophils in the bone marrow **(A)**, the markers of positive migration and aging in the bloodstream **(B)**, and the markers of migration to various tissues and organs in the human body **(C)** (Created using Figdraw).

The diverse functional responses of neutrophils are triggered by the changes in transcriptional activation and the expression or activity of surface molecules. These phenotypic variations are often detectable only in a subset of neutrophils, indicating a significant heterogeneity among neutrophils (13, 16). Therefore, the heterogeneity of neutrophils in different states can be observed through the differential expression of certain markers (Figure 1). Immature neutrophils, which are in the early stages of differentiation, are primarily located in the bone marrow and can be rapidly mobilized into the peripheral circulation in response to specific stimuli (17). CD33, CD15, CD11b, CD16, and CD10 are the signature molecules of immature differentiated neutrophils (18–20). Mature neutrophils are cells that have completed the differentiation process and possess the ability to perform immune defense functions. They carry a range of preformed adhesion and chemotactic receptors, as well as effector proteins, enabling them to migrate rapidly and respond to a variety of microbial and sterile stimuli (17). Phenotypes such as CD16hi, CXCR2^hi^, CXCR4^low^, and CD62L^hi^ are characteristics of mature circulating neutrophils (21). The down-regulation of the chemokine receptor 4 (CXC chemokine receptor 4, CXCR4) is a vital event in the mobilization of neutrophils from the bone marrow (22), and chemokines (including CXCL8, CXCL1, CXCL2, and CXCL5) can stimulate the release of neutrophils into the circulation and their migration to inflammatory sites via CXCR2 signaling, where they adhere to the endothelium (23–26).

Neutrophil activation by inflammatory signals initiates transendothelial migration into tissues. Interactions with selectins and chemokines lead to vesicle secretion and exposure of membrane proteins. Activated neutrophils up-regulate CD11b/CD18, enhancing firm adhesion to endothelium (3). The activation of neutrophils also results in an increase in the expression of CD66b, which can be mobilized from intracellular granules to the cell membrane in the process of degranulation (27). Moreover, in the context of infection, the expression of CD64 on the surface of neutrophils is rapidly upregulated (under normal conditions, CD64 is barely expressed on neutrophil surfaces). This upregulation serves as one of the early responses of neutrophils to pathogen invasion and is regarded as a crucial step in the innate immune response (28, 29). The CD177 glycoprotein (NB1) is expressed exclusively on the surface of human neutrophils and controls transendothelial transport through interaction with platelet endothelial cell adhesion molecules (30).

Aging neutrophils, in addition to being smaller in size and containing fewer granules (31), exhibit the enhanced pro-inflammatory activity but have impaired adhesion and phagocytic functions (32, 33). The general phenotype of “aged neutrophils” includes CXCR2^low^, CXCR4^hi^, CD62L^low^, CD11b^hi^, CD11c^hi^ and CD47^low^ (31, 34–37). Chemokine CXC motif ligand 12 (CXCL12) and its receptor CXCR4 are widely expressed in different cells in human bone marrow, heart, and other tissues (38). CXCR4 is generally considered to be the sole specific receptor for CXCL12, and the multi-pathway signaling of CXCR4 can mediate different biological movements and responses including cell migration, chemotaxis, proliferation, anti-apoptosis, homing, and adhesion (39). The retention and release of neutrophils in the bone marrow are controlled by the synergistic action of the CXCL12/CXCR4 axis (35, 40). In the peripheral blood, aged neutrophils with increased CXCR4 expression home to the bone marrow through the chemokine CXCL12 pathway, and once in the bone marrow, CD62L^low^ neutrophils migrate towards macrophages, causing the clearance of these neutrophils from the bloodstream (31, 35, 36, 38). Moreover, the reduced expression of the “do not eat me” molecule CD47 can enhance the recognition and phagocytic action of macrophages (34, 37). Other markers associated with aged neutrophils include CD45, CD24, ICAM-1 and TLR4 (41–43).

In addition to the bloodstream, under normal conditions, neutrophils can also migrate to various tissues and spontaneously change their phenotype. For instance, the spleen exhibits neutrophils with the phenotypeCD62L^low^CD11b^hi^ICAM1^hi^ (44), and the neutrophils located in the marginal zone of the human spleen are referred to as “neutrophil B cell helpers” (NBH cells) (45). This subpopulation (CD15int/lo, CD16int/low, and CD11b^hi^) can release B cell-stimulating molecules, including B cell-activating factor (BAFF) and CD40 ligand (CD40l), promoting the secretion of IgM and IgG. Prostates express neutrophils with the phenotype CD11b ^+^ Ly6G ^+^ Ly6C^lo^, possessing stronger immunosuppressive activity (46). Currently, our understanding of how neutrophils change into various phenotypes in different tissues and what roles they play is still limited, and there is a vast unknown territory awaiting our continued exploration.

After migrating to tissues, neutrophils can return to the bloodstream, a process known as reverse transendothelial migration (rTEM). Markers associated with reverse migrating neutrophils include CD18, ICAM1 (47), CXCR4^low^ (48), and neutrophil elastase (NE), which degrade junctional adhesion molecule-C (JAM-C) (49). CD18 can interact with endothelial cell surface molecules (including binding to ICAM-1) (50, 51), and the homing of neutrophils to the bone marrow is mediated by CXCR4 (52). Neutrophil rTEM regulates the proliferation of T cells and B cells, as well as NET formation, and induces systemic inflammation and interaction with the immune system by clearing excess neutrophils from local tissues (49). Therefore, targeting neutrophil rTEM may offer a novel therapeutic strategy for resolving inflammation.

### 2.3 Cardiac biological functions of neutrophils

#### 2.3.1 Generation and chemotaxis

Healthy adults need to produce 10^11^ neutrophils daily, and mice require 10^7^ (14). Under homeostatic conditions, the number of mature neutrophils remains relatively stable and can be released into circulation from the bone marrow following a circadian rhythm through the mutual regulation of CXCR2/CXCR4 and the interaction with CXCL1 and CXCL2 produced by endothelial cells and megakaryocytes (53–55). However, in acute inflammation and emergency situations including myocardial infarction, the production of neutrophils can be accelerated and maintained by proliferative signals from cells subjected to ischemic injury or exposed to ischemic damage, and their numbers can rapidly increase in circulation (56). On the one hand, they can phagocytose dead cells and cellular debris, clearing sterile inflammation; on the other hand, they can also send signals to recruit monocytes, promoting the phagocytosis of apoptotic or necrotic neutrophils (57). Meanwhile, after recognizing microbes and/or inflammatory stimuli, neutrophils can immediately migrate from circulating blood to the site of inflammation through an adhesion/migration cascade, exerting their effector functions and responding to damage by capturing and eliminating pathogens through phagocytosis, producing ROS, degranulation, secreting vesicles, and forming NETs (58). The activated neutrophils can up-regulate OXPHOS genes, relying on mitochondrial respiration to produce sufficient ATP to ensure the continuation of cell migration and chemotaxis (59, 60).

#### 2.3.2 Pro-inflammatory and repair functions

Neutrophils display distinct phenotypes and functions during pro- and anti-inflammatory processes. Post-cardiac injury, they polarize temporally from pro-inflammatory “N1” to reparative “N2” phenotypes. In myocardial infarction's early phase, neutrophils activate and polarize into N1 via pattern recognition receptors (PRRs) recognizing pathogen-associated molecular patterns (PAMPs) or damage-associated molecular patterns (DAMPs), enhancing pro-inflammatory functions (61, 62). On the first day, N1 type neutrophils produce and release pro-inflammatory mediators, including cytokines (such as TNF-α, IL-1β) and chemokines (like CCL3), further recruiting other immune cells (63). Simultaneously, polarization also occurs in the peripheral blood and bone marrow of myocardial infarction models, which may involve the reverse transport of neutrophils, stimulating granulopoiesis and spreading inflammation (64). During the resolution of inflammation and tissue repair, N2 type neutrophils are dominant (65, 66), expressing anti-inflammatory markers from the 5th to the 7th day of injury, which assists in the resolution of inflammation and the clearance of apoptotic myocardial cells (67). Interestingly, neutrophils also promote cardiac repair through the production of matrix proteins required for fibrotic scar formation (including fibronectin and fibrinogen) and by secreting MMP9 to promote angiogenic responses (68).

#### 2.3.3 Immune regulation

Neutrophils can also interact with various immune cells, which include dendritic cells, T cells, and B cells, and modulate their functions (69). For instance, conventional neutrophils in the marginal zone, known as NBH cells, are thought to facilitate B cell proliferation and antibody production (45). The interaction between neutrophils and dendritic cells can influence the maturation and antigen-presenting capacity of dendritic cells, thereby affecting the activation and differentiation of T cells (70). Neutrophils are also capable of expressing and secreting molecules that affect T cell functions, such as arginase 1 and inducible nitric oxide synthase (iNOS), which can hinder T cell proliferation and activation, thus regulating T cell-mediated immune responses to some extent (71). These interactions suggest that neutrophils may exert a more complex role in forming and regulating adaptive immune responses than previously thought. In addition, neutrophils modulate immune responses by secreting extracellular vesicles (EVs) that contain microRNAs (miRNAs), Specific miRNAs, such as miR-130a and miR-155, are known to regulate various stages of neutrophil development and function (72).

## 3 Neutrophil death mechanisms

**Figure 2:**
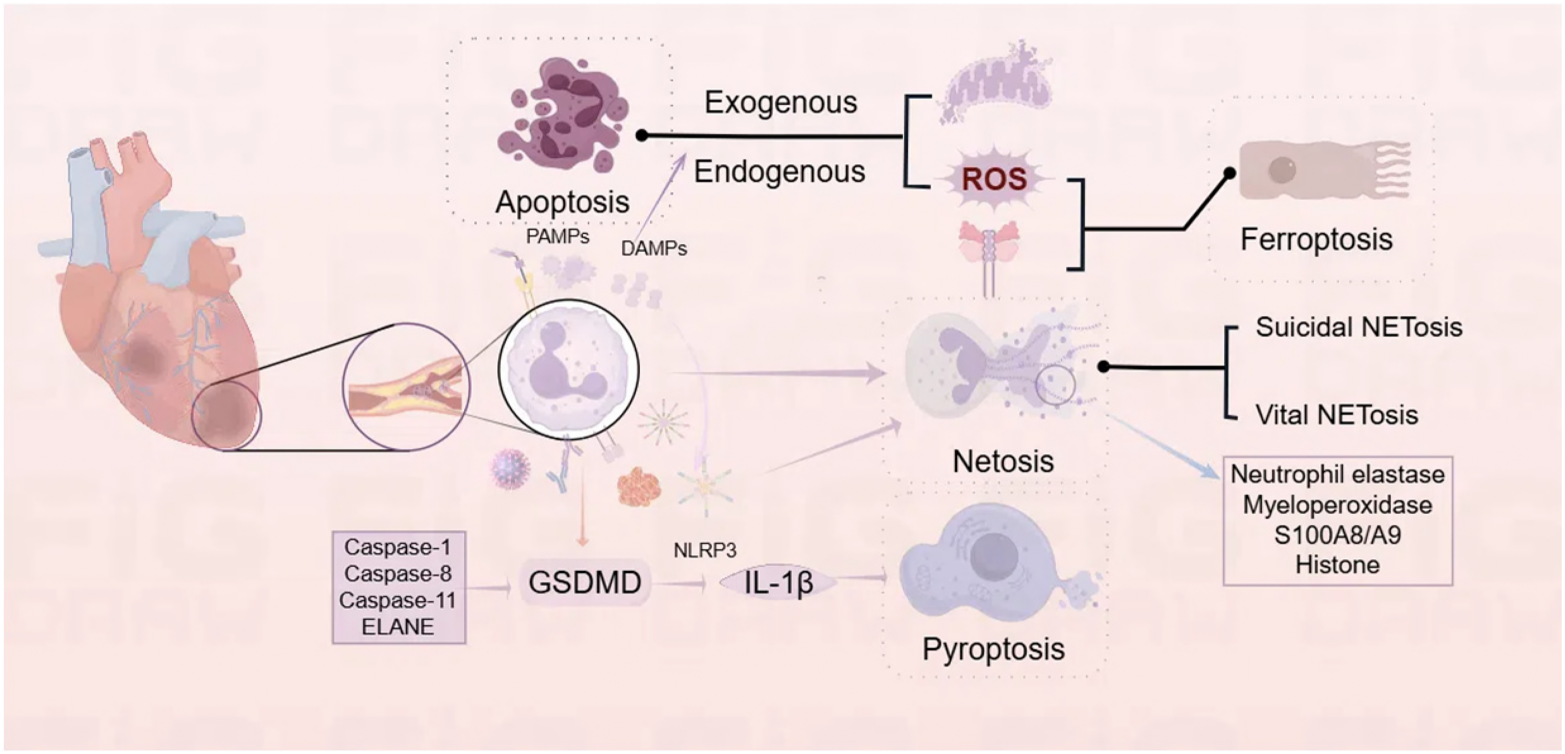
Mechanisms of neutrophil death. provides an overview of the four common modes of neutrophil death: apoptosis, NETosis, pyroptosis, and ferroptosis. At sites of cardiac injury, neutrophil death can be triggered by a variety of factors, including pathogen-associated molecular patterns (PAMPs), damage-associated molecular patterns (DAMPs), bacteria, and viruses. Different modes of death can be induced through distinct pathways and are interrelated (Created using Figdraw).

Neutrophils can undergo various modes of death, such as apoptosis, NETosis, pyroptosis, and ferroptosis (Figure 2). Understanding the diverse death mechanisms of neutrophils can further elucidate the critical points at which neutrophils are involved in the pathogenesis of CVDs.

### 3.1 Apoptosis in neutrophils

The maturation of neutrophils reduces the transcription and translation rates, thereby decreasing the expression of anti-apoptotic proteins while retaining that of pro-apoptotic effector proteins (73). Under normal conditions, neutrophils enter the circulation and migrate to peripheral tissues after maturation. A subset of neutrophils, after fulfilling their physiological functions in the tissues (such as destroying the invading organisms), undergo “spontaneous apoptosis” through a series of orderly, programmed intracellular processes, ultimately leading to cell death. This form of cell death maintains the integrity of the neutrophil membrane, preventing the release of intracellular toxic substances, and limiting their destructive capacity to surrounding tissues (74, 75). It is crucial for maintaining the balance of circulating neutrophil numbers and the pro-inflammatory/anti-inflammatory balance in the body (14). Following myocardial ischemia, cardiomyocytes subjected to severe ischemia and hypoxia may suffer injury or even necrosis, leading to the release of DAMPs (76). These DAMPs can interact with PRRs to trigger intercellular signaling cascades, promoting the expression of pro-inflammatory cytokines and chemokines (77). This process further amplifies inflammation and exacerbates myocardial injury (78, 79). Neutrophil apoptosis is one of the essential mechanisms for maintaining homeostasis and limiting inflammatory damage. Apoptosis of neutrophils is initiated through two distinct signaling pathways, namely, the intrinsic and extrinsic pathways. The extrinsic apoptosis pathway in neutrophils depends on ROS produced by NADPH oxidase (80), and its mechanism may be related to the ability of ROS to directly change the activity of intracellular signaling pathways involved in neutrophil death/survival, such as NF-kB and MAPK (81, 82). The intrinsic apoptosis pathway is mediated by mitochondria, which is featured with the loss of mitochondrial membrane potential and the release of pro-apoptotic factors like cytochrome c into the cytosol (83, 84).

### 3.2 NETosis

Neutrophils are a vital component of the innate immune system. Apart from phagocytosing and destroying invading microbial pathogens and releasing antimicrobial granules, they can also exert their functional effects in tissues through various mechanisms, including the production and release of reactive oxygen species (ROS) and neutrophil extracellular traps (NETs), causing damage and destruction of pathogens (85–87). At first, NETs were described by Brinkmann et al. in 2004 (88) and are the extracellular web-like structures released by activated neutrophils, with DNA as its backbone and combined with various antimicrobial proteins including histones, neutrophil elastase (NE), calprotectin B (also known as S100A9), and myeloperoxidase (MPO), which primary function is to capture and kill microorganisms such as bacteria, fungi, and viruses (89). NETs can also participate in the onset and progression of CVDs by promoting thrombosis and exacerbating inflammatory responses, among other mechanisms (90). The activated neutrophils produce and trigger a special form of cell death called NETosis when they generate and release NETs. NETosis is distinct from both apoptosis and necrosis and is entirely independent of the action of caspases involved in apoptosis. Although the lifespan of neutrophils is short, the function of released NETs can last for several days to further exert their effects. Neutrophils can be activated by recognition through Toll-like receptor 2 (TLR2), Toll-like receptor 4 (TLR4), and complement receptors (91), and they are stimulated by pathogenic microorganisms, activated platelets, specific cytokines (such as IL-8 and TNF-α), lipopolysaccharides (LPS), and other stimuli (92), finally activating the NADPH oxidase (93). NADPH oxidase is one of the major sources of intracellular ROS, which can activate a series of signaling pathways. ROS can activate protein arginine deiminase 4 (PAD4) (94) and it catalyzes histone citrullination, such as converting arginine in histone H3 to citrullinated H3 (CitH3), leading to chromatin decondensation (95). ROS can also activate the translocation of NE and MPO from azurophilic granules to the nucleus. Subsequently, NE cleaves histone H1 and modifies core histones, ultimately resulting in the release of chromatin into the extracellular space to form NETs (96). Additionally, some viruses, including influenza A, HIV-1, and encephalomyocarditis virus, can also trigger the formation of NETs (97–99), but not all pathways that result in the formation of NETs can result in neutrophil death. Currently, two different forms of NETosis have been identified, including the classical or suicidal NETosis (which lead to cell death through the formation of NETs with nuclear membrane rupture) and “vital NETosis” or non-suicidal NETosis (which maintains cell viability and a range of effector functions, and is mediated by vesicles) (100, 101). Non-suicidal NETosis is thought to play a more significant role in regulating pathogen infection. Although NETs are vital effectors in clearing bacterial infections and sterile inflammation, they can also cause tissue damage (102–104). S100A8/A9, belonging to the S100 family of calcium-binding proteins, exerts a certain effect on various inflammatory diseases (105). When NETs are generated, S100A8/A9 can be released from the cytoplasm of neutrophils, activating surrounding immune cells and thus amplifying the inflammatory response (106). Therefore, inhibiting the overactivation of the innate immune system remains a key therapeutic approach. In addition, miRNAs can also regulate the formation of NETs. For example, miR-155 modulates NET formation by targeting PAD4 (107), while exosomes containing miR-505 have been shown to promote NET formation (108). Understanding the complex interplay among miRNAs, NETs, and cardiovascular diseases is an active area of research that may lead to the discovery of novel therapeutic strategies.

### 3.3 Pyroptosis of neutrophils

Pyroptosis is a form of regulated cell death mediated by the Gasdermin family of proteins, which is characterized by the formation of membrane pores, causing cell swelling and rupture, release of inflammatory mediators, and promotion of inflammatory responses (109). Pyroptosis has been identified as another mode of regulated cell death that can occur in mature neutrophils, and it is triggered by various signaling pathways, including the canonical (caspase-1 dependent) [NOD-like receptor family pyrin domain containing 3, NLRP3] and non-canonical (caspase-11 dependent) inflammasomes [such as Absent in Melanoma 2, AIM2], death receptors (RIPK1 and caspase-8), and granule serine proteases (110). Neutrophils can express Gasdermin D (GSDMD) and maintain their ability to release IL-1β (a pro-inflammatory cytokine) through a GSDMD-dependent pathway, thereby promoting inflammatory responses and immune responses without cell death (111). As a key molecule in regulating cell pyroptotic necrosis, the absence of GSDMD reduces the degree of myocardial fibrosis after myocardial infarction, and decreases IL-1β released by infiltrating myeloid cells in the heart, thus playing a cardioprotective role (109). This result indicates that GSDMD is a potential target for improving ventricular remodeling and reducing heart failure (HF) after AMI. Moreover, NETosis may be related to the pyroptosis of neutrophils (111). The NLRP3 inflammasome can recognize PAMPs and DAMPs, and the activation of the NLRP3 inflammasome can stimulate NETosis, further amplifying the inflammatory response (112). The GSDMD inhibitor Disulfiram (DSF) can inhibit the release of NETs (113), suggesting that different forms of neutrophil death are interconnected and collectively affect the body's immune function.

### 3.4 Ferroptosis of neutrophils

Ferroptosis, first proposed by Stockwell et al. (114), is a novel form of regulated cell death caused by iron overload and the accumulation of lipid ROS, accompanied by DAMPs. Implicated in inflammation and disease pathogenesis, it is increasingly linked to cardiovascular diseases (CVDs). Neutrophil ferroptosis has become a burgeoning research area. Elevated lipid ROS and neutrophil ferroptosis have been observed in the serum of systemic lupus erythematosus (SLE) patients (115), yet there have been no reports on neutrophil ferroptosis in CVDs. However, it is believed that further exploration of neutrophil ferroptosis may provide new opportunities for the treatment of CVDs.

## 4 Neutrophils and CVDs

### 4.1 Neutrophils and atherosclerosis (AS)

The development of AS relies on a chronic inflammatory process driven by lipids, from the initial stage of vulnerable atherosclerotic plaques to their eventual rupture, where neutrophils play a key pathophysiological role. Megen et al. reported (116) the presence of neutrophils and NETs in atherosclerotic lesions in mice and humans. Initially, hyperlipidemia can induce neutrophilia (117), and neutrophils can damage endothelial function by mediating the deposition of granule proteins on endothelial cells, inducing adhesion, and promoting the recruitment of monocytes, thereby exacerbating the local response in the atherosclerotic process (118). Meanwhile, activated mast cells present in the intima and perivascular tissue of atherosclerotic plaques can release chemokines (such as CXCL1), inducing further recruitment of neutrophils to the inflammatory site (119). In AS mouse models, sterile inflammation is also shown to drive cytokine production, and neutrophils, once activated, initiate effector functions and release NETs (8). Research by Knight et al. (120) showed that chloromethyl ketone inhibited PAD4, thereby reducing the formation of NETs and the lesion area of atherosclerotic plaques, further suggesting the important role of NETs in AS. Studies have indicated (121) that human neutrophil granules express bactericidal/permeability-increasing protein (BPI), which may participate in AS and atherosclerotic thrombosis, highlighting AS as an inflammatory and immune response. While lipid-lowering therapy is fundamental in AS treatment, it is insufficient for mitigating residual cardiovascular risk, suggesting that targeted neutrophil therapy could be a promising future approach.

### 4.2 Neutrophils and acute coronary syndrome (ACS)

ACS is a group of clinical syndromes caused by a sudden reduction in blood supply to the heart, encompassing Non-ST-segment Elevation Myocardial Infarction (NSTEMI), ST-segment Elevation Myocardial Infarction (STEMI), and Unstable Angina (UA) (122). In ACS, neutrophils are involved in all aspects, including the activation of the early inflammatory response, myocardial cell necrosis, and the later stage of myocardial scar repair. The neutrophil count is regarded as an independent prognostic factor for patients with ACS (123). Neutrophils are present in the early vulnerable ruptured/eroded atherosclerotic plaques and are massively recruited in the late stage of plaque progression (124). After myocardial infarction, a large amounts of DAMPs are produced by the damaged myocardial cells (125), including the nuclei of myocardial cells (such as HMGB1), cytoplasm (such as RNA), extracellular matrix (such as fibronectin), mitochondria (such as mtDNA), and contractile components (myosin) (126, 127), which triggers the sterile inflammatory response after myocardial infarction and ultimately activates a complete innate immune signaling pathway (128). Numerous neutrophils are activated and immediately initiate a series of processes, including neutrophil recruitment, adhesion, binding, and final migration and infiltration (129). Activated neutrophils can induce endothelial cell damage via apoptosis or protease production. Proteases disrupt endothelial adhesion to the vascular wall, leading to cell detachment, exposure of the underlying connective tissue matrix, platelet adhesion, and thrombosis (130). In the meantime, thrombin-activated platelets interact with neutrophils at the site of plaque rupture, leading to local NETosis and the activation of tissue factor (131). After myocardial infarction, neutrophils can also release S100A8/A9 in the infarction area through NETosis, interact with Toll-like receptor (TLR) 4 on naïve neutrophils, activate the NLRP3 inflammasome of the NOD-like receptor (NLR) family pyrin domain-containing 3, and amplify granule production through IL-1 and IL-18 dependent signaling pathways (132), eventually promoting the development of myocardial fibrosis (133). Subsequently, the recruitment of neutrophils can not only clear cellular debris and dead cells, but also produce key molecular signals for monocyte/macrophage reprogramming (129). Neutrophils release MMP8 and MMP9, degrading the extracellular matrix. The inhibition of MMP-9 in mice can reduce left ventricular dilation and collagen accumulation in the infarction area after coronary artery ligation (134), further proving that neutrophils are indispensable in adverse cardiac remodeling after myocardial infarction (135).

### 4.3 Neutrophils and myocardial ischemia/reperfusion injury (MI/RI)

Reperfusion of ischemic myocardium is an absolute necessity to save the myocardial tissue from ultimate death. The activation of neutrophils and the accumulation of ROS are the important pathophysiological mechanisms that mediate MI/RI. Within 24–48 h after myocardial reperfusion, neutrophils are the main infiltrating cells (136), which can exacerbate myocardial damage by mediating inflammatory responses and affecting microcirculatory blood flow. The primary mechanism involves the activation of nicotinamide adenine dinucleotide phosphate (NADPH) oxidase (respiratory burst), releasing a large amount of ROS (137). ROS can directly react with lipids, proteins, and DNA, generating cellular damage and inducing the release of cytokines and chemokines that are partially mediated by NF-κB. In the myocardial ischemia-reperfusion (MI/R) model, blocking NF-κB can reduce myocardial injury and left ventricular remodeling (138). DAMPs released during MI/R can activate the complement cascade, which up-regulates adhesion molecules on endothelial cells to promote neutrophil migration (139). PDE4B plays a key role in MI/RI by mediating neutrophilic inflammatory responses and microcirculatory disturbances. Blocking PDE4B can not only reduce myocardial cell death, but also improve coronary microvascular obstruction, which provides the dual cardiac protection (140), and offers a novel perspective for preventing and treating MI/RI. The development of subtype-selective PDE4B inhibitors or the repurposing of existing PDE drugs may become a new cardiac protective agent for patients with acute myocardial infarction during reperfusion.

### 4.4 Neutrophils and hypertension (HTN)

In addition to neuro-humoral regulatory mechanisms, immune-inflammatory responses exert a critical role in the pathological cardiac remodeling caused by HTN. Evidence has suggested that the innate immune system has a vital role in the development of hypertension since the 1960s (141). Morton et al. (142) demonstrated neutrophils' direct role in blood pressure regulation. These cells infiltrate arteries, heart, and kidneys, potentially promoting fibrosis through secretion of pro-oxidant, pro-inflammatory, and profibrotic molecules, and stimulating immune cell infiltration into tissues such as blood vessels and the heart (143), and leading to inflammation and fibrosis related to HTN. In patients with HTN or animal models of HTN, neutrophils isolated from peripheral blood exhibit high levels of ROS (144, 145), which enhances their oxidative stress characteristics and triggers multiple processes such as inflammation, proliferation, and fibrosis, thereby damaging vascular function. Previous studies have suggested that an increased neutrophil count and an elevated neutrophil/lymphocyte ratio (NLR) in hypertensive patients are correlated with an increased risk of hypertension (146), with NLR being higher in non-dipper hypertensive patients. Additionally, some clinical studies have shown that the use of certain antihypertensive medications can reduce the NLR in patients with HTN.

### 4.5 Neutrophils and atrial fibrillation (AF)

AF is the most commonly seen clinical arrhythmia. Patients with AF typically exhibit an increased inflammatory signal activity (147). In both patients with AF and in angiotensin II-induced AF mouse models, increased infiltration of immune cells such as neutrophils, macrophages, and monocytes in the atrial tissue can be detected (148–150). Even in the absence of underlying structural heart disease, leukocytes can be found in the atrial tissue of patients with AF (151). Lipid inflammatory mediators such as platelet-activating factor (PAF), which are synthesized by activated neutrophils, can induce atrial and ventricular arrhythmias as well as repolarization abnormalities in isolated cardiomyocytes (152). The sustained rapid pacing can lead to the loss of myocardial cell structure, which provides a strong stimulus for the secretion of NETs. NETs can promote apoptosis in cardiomyocytes via the autophagy pathway, and cause swelling of mitochondria in cardiomyocytes, depolarization of mitochondrial membrane potential, and increased production of mitochondrial ROS (153), resulting in an increased susceptibility to AF (152). In the *in vivo* experiments, the degradation of NETs by DNase I can reduce the duration of AF and improve fibrosis associated with AF, providing a new strategy for the treatment of AF (153). An elevated NLR is not only seen in patients with AF accompanied by underlying structural heart disease and in patients undergoing paroxysmal AF, but can also act as a predictive marker for the recurrence of AF after cardiac conversion.

### 4.6 Neutrophils and heart failure (HF)

HF is a complex clinical syndrome caused by various etiologies that lead to structural and/or functional abnormalities of the heart, resulting in impaired ventricular systolic and/or diastolic function (154), Ischemic heart disease is the most common cause of heart failure (155). Although the role of neutrophils as first responders in the acute phase of myocardial infarction to clear dead cells, their role in chronic ischemic heart failure remains unclear (135). The relationship between neutrophils and HF is multifaceted, involving inflammation, cardiac remodeling, and immune regulation. Hemodynamic changes in HF can induce a sterile inflammatory state (156). Early inflammatory responses include the migration of neutrophils and macrophages, production of pro-inflammatory cytokines and chemokines such as TNF-α, IL-1, and IL-6 at different stages of HF (157), mediation of myocardial cell necrosis and exacerbation of adverse cardiac remodeling (158), making the targeted inhibition of neutrophil-derived pro-inflammatory factors an active area of research for treating HF. Studies have shown that in the HF mouse of myocardial infarction models, the depletion of neutrophils through antibody-mediated or genetic methods can halt the progression of HF, especially the left ventricular remodeling and fibrosis progression in the middle and late stages of HF (135), further indicating that neutrophils are a potential therapeutic target for lowering myocardial damage and preventing HF (159, 160). Neutrophils may also directly damage cardiomyocytes by releasing ROS and proteolytic enzymes, leading to cardiac dysfunction (161). The NLR also has predictive value in the severity, prognosis, and diagnosis of HF, with its increased level being associated with a poor prognosis in patients with HF (162).

### 4.7 Neutrophils and viral myocarditis (VMC)

Myocarditis can be caused by various infectious and non-infectious etiologies, such as viruses, immune system activation (e.g., autoimmune diseases like sarcoidosis or immune stimulation due to vaccines or cancer therapies), or exposure to toxins and medications. Among infectious causes, viruses are the most common etiology. The localized or diffuse inflammatory lesions of the myocardium caused by viral infection are referred to as VMC, with Coxsackie virus B3 (CVB3) being a major pathogenic agent (163). Molecules derived from the virus, which act as PAMPs, are effective inducers of NETs. The role of neutrophils in virus-associated diseases is not previously emphasized, but research by Fairweather et al. (164) reported that the severity of acute myocarditis was closely associated with the accumulation of neutrophils in the heart. Patients and mice with CVB3-induced myocarditis have significantly increased levels of S100A8/S100A9, which exacerbate oxidative stress and viral replication in myocarditis (165, 166), further indicating the important role of neutrophils in the antiviral immune response (167). In the acute phase of CVB3-induced VMC in mice, Vγ1γδT cells are the main infiltrating cells with cardioprotective effects. However, with the depletion of Vγ1γδT cells, the infiltration of NK cells, macrophages, and neutrophils into the heart gradually increases (168). Neutrophils mainly recognize CVB3 via endosomal TLR-8, thereby triggering NF-κB activation and inducing the release of NETs (169), and neutrophil depletion mediated by anti-Ly6G antibodies or PAD4-dependent NETs blockade can reduce cardiac injury and leukocyte recruitment (170). Virus internalization increases cell survival, up-regulates CD11b expression, enhances adhesion to fibrinogen and fibronectin, and boosts secretion of IL-6, IL-1β, TNF-α, and IL-8, thereby promoting neutrophil migration to target organs and viral spread (169). Consequently, neutrophils play both direct and indirect roles in the pathogenesis of CVB3-induced VMC.

## 5 Research progress on cardiac remodeling treatment strategies targeting neutrophils

**Figure 3:**
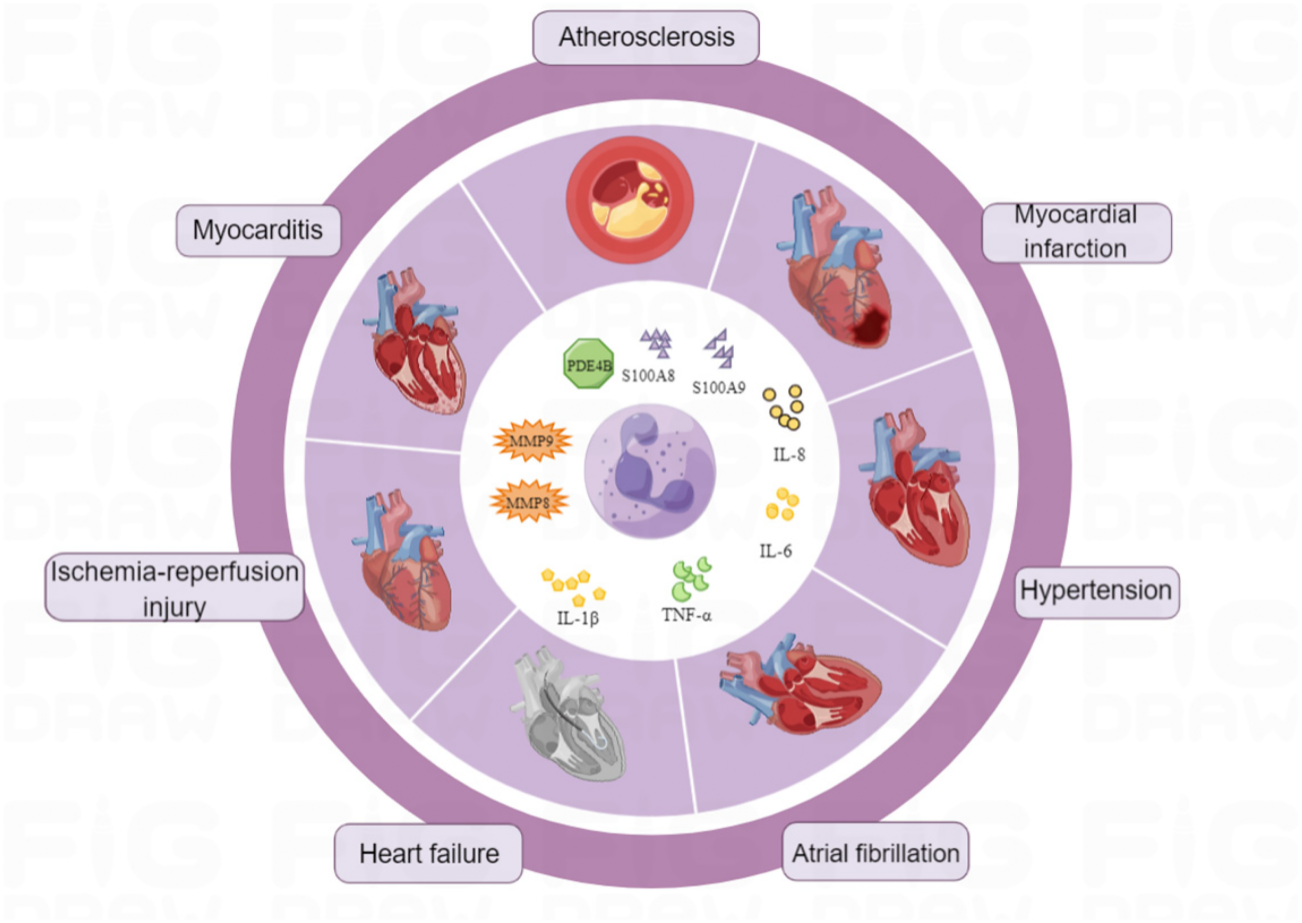
Neutrophils and cardiovascular diseases. Illustrates the complex relationship between neutrophils and cardiovascular diseases. Neutrophils play a pivotal role in the pathogenesis of various cardiovascular conditions, including atherosclerosis, myocardial infarction, and heart failure. They contribute to inflammation, plaque formation, and tissue damage through the release of pro-inflammatory cytokines, reactive oxygen species, and proteolytic enzymes.

At present, there are no widely used clinical standard drugs. Some studies have indicated that non-steroidal anti-inflammatory drugs (NSAIDs) can decrease the activation and aggregation of neutrophils, decreasing their role in the inflammatory response after myocardial infarction (171). Drug treatment strategies targeting neutrophils in CVDs are under development. These strategies may modulate neutrophil behavior (activation, recruitment, migration, infiltration), reduce NETs production, boost phagocytosis, and thus, prevent and treat CVDs (Figure 3).

### 5.1 Inhibition of neutrophil activation and adhesion

Stamp2 prevents maladaptive structural remodeling and contractile dysfunction of the heart after ischemia by diminishing neutrophil activation (172). RTP-026 selectively activates formyl-peptide receptor type 2 (FPR2), which can reduce the activation of neutrophils and monocytes, thereby decreasing the infarct size (173). Tetrandrine (TTD), isolated from the traditional Chinese medicinal herb Stephania tetrandra, has been shown to mitigate MI/RI through the inhibition of neutrophil activation, thereby reducing inflammatory responses and the production of ROS (174). This provides a novel strategy for cardiac protection, namely, the clinical targeting of neutrophil activation to mitigate inflammatory responses and tissue damage, thereby improving patient outcomes.

### 5.2 Reduction of neutrophil migration and infiltration

Evasin-3 is a CXC chemokine-binding protein that can bind to CXCL1, which can prevent its interaction with the receptor CXCR2, thereby inhibiting the biological activity of CXCL1. This reduction in neutrophil migration and infiltration into inflammatory sites may help to lower the risk of AS-related diseases (175). The nicotinamide phosphoribosyl transferase (NAMPT) inhibitor FK866 can decrease the production of CXCL1 by endothelial cells, reduce the recruitment of neutrophils to ischemic myocardium, and subsequently alleviate inflammation in atherosclerotic plaques (176). CXCL8 recruits neutrophils through the PI3K/AKT pathway, and curcumin reduces neutrophil recruitment by inhibiting this pathway, thereby mitigating inflammatory responses (177). In a mouse model of myocardial ischemia, administration of anti-CCL5 monoclonal antibodies significantly decreases the infarct size and lowers the risk of HF development, which is associated with reduced neutrophil recruitment in the infarcted heart (178). In an animal model of myocardial reperfusion, sgp130Fc reduces the infiltration of neutrophils and macrophages into the myocardium, decreasing infarct size and preserving cardiac function (179). Necrosulfonamide (NSA) reduces the infiltration of neutrophils into the cardiac injury area by blocking the formation of pores by GSDMD on the cell membrane, thus reducing the occurrence of pyroptosis (109). Colchicine is an ancient anti-inflammatory drug that has been used for different rheumatic and immune diseases for many years. Recently, an increasing number of scholars have found that colchicine exerts a vital role in the treatment of CVDs (180). One of the mechanisms is that colchicine can inhibit microtubule polymerization by binding to tubulin dimers and negatively regulate the migration and infiltration of neutrophils after injury (181). Basic research indicates that therapeutically targeting neutrophil migration and infiltration can protect the heart, reduce infarct size, enhance cardiac function, and potentially improve patient outcomes.

### 5.3 Reduction of NETs formation

Inhibiting the protein components related to NETs, including MPO, PAD4, and CitH3, may also be one of the intervention methods. Nitroxides are a class of reversible MPO inhibitors that suppress the production of HOCl by serving as substrates for the MPO compound I, reducing the release of NETs (182). MPO inhibitors also include 4-aminobenzoic acid hydrazide (182) and PF1355 (183), which can not only reduce neutrophil aggregation but also decrease the formation of NETs. Chloro-amidine, by inhibiting PAD4, can not only reduce NETosis but also inhibit the recruitment of neutrophils and macrophages to inflammatory sites, reducing the size of atherosclerotic lesions (184). DNase is a commonly used NETs inhibitor, Brinkmann et al. (88) first demonstrated that the addition of DNase I *in vitro* degraded NETs. Basic research also shows that both DNase I and chloro-amidine can provide additional AS protection through inhibiting NETosis in mice (8). However, the mechanism of action of DNase I does not prevent the production of NETs but rather decompose the structure of NETs (185), so its efficacy is still a matter of debate. In the MIRI model, MKEY treatment reduced the inflammatory response after MIRI, decreased the infarct size, and also reduced the levels of CitH3 in the infarcted tissue, indicating that MKEY can prevent the formation of NETs in the body (186). The use of S100A8/A9 blocker ABR-238901 immediately after myocardial infarction can reduce the number of neutrophils in the myocardium and the presence of S100A9, reducing the size of myocardial infarction (187), and having a positive impact on cardiac injury. The development of new neutrophil-targeting therapeutic drugs and compounds targeting NETs-associated protein components may represent a change in the current treatment status of CVDs. Moreover, a meta-analysis showed that miRNAs have the potential to serve as a therapeutic target for inhibiting NETosis, and limiting a variety of clinical diseases (188). Targeting NETs-associated protein components or using miRNAs as therapeutic tools to regulate NETs formation may find broad applications in the treatment of cardiovascular diseases in the future.

### 5.4 Enhancement of neutrophil phagocytic capacity

Treatment of rats with acute myocardial infarction using the FPR2 agonist BMS-986235 enhances the phagocytic capacity of neutrophils, aiding in the clearance of dead cells and inflammatory mediators, lowering inflammatory damage, and enhancing adverse remodeling and cardiac function post-myocardial infarction (189). Future therapies may combine multiple aspects of immunomodulation to achieve additional benefits, while other challenges such as the targeting of inflammation in cardiovascular disease patients must also be considered.

## 6 Conclusions and perspectives

In summary, neutrophils, as key immune cells, play a crucial role in CVD treatment. They interact with both innate and adaptive immune cells under inflammatory conditions, contributing to chronic sterile inflammation in cardiovascular patients and the progression of diseases such as AS, ACS, MI/RI, HNT, AF, HF and VMC. Current research emphasizes neutrophil activation, recruitment, and NETs production, which could inform therapeutic strategies targeting neutrophils for the prevention and treatment of CVDs. It is worth noting that the inconclusive results of current anti-inflammatory drug trials in clinical settings highlight an incomplete understanding of the complex inflammatory networks in CVDs. Moreover, the use of NET inhibitors currently focus more on animal experiments, and in-depth research is lacking in clinical trials. Against the backdrop of the coexistence of risks and benefits in current anti-inflammatory treatments, further exploration of highly selective targeted drugs to avoid affecting the normal functions of neutrophils is a subject deserving further investigation.
